# High Dose Atorvastatin Associated with Increased Risk of Significant Hepatotoxicity in Comparison to Simvastatin in UK GPRD Cohort

**DOI:** 10.1371/journal.pone.0151587

**Published:** 2016-03-16

**Authors:** Alan T. Clarke, Paul C. D. Johnson, Gillian C. Hall, Ian Ford, Peter R. Mills

**Affiliations:** 1 Dept. of Gastroenterology, Queen Elizabeth University Hospital, Glasgow, United Kingdom; 2 Robertson Centre for Biostatistics, University of Glasgow, Glasgow, United Kingdom; 3 Grimsdyke House London, London, United Kingdom; 4 Dept. of Gastroenterology, Gartnavel General Hospital, Glasgow, United Kingdom; 5 Institute of Biodiversity, Animal Health and Comparative Medicine, University of Glasgow, Glasgow, United Kingdom; University of Pisa, ITALY

## Abstract

**Background & Aims:**

Occasional risk of serious liver dysfunction and autoimmune hepatitis during atorvastatin therapy has been reported. We compared the risk of hepatotoxicity in atorvastatin relative to simvastatin treatment.

**Methods:**

The UK GPRD identified patients with a first prescription for simvastatin [164,407] or atorvastatin [76,411] between 1997 and 2006, but with no prior record of liver disease, alcohol-related diagnosis, or liver dysfunction. Incident liver dysfunction in the following six months was identified by biochemical value and compared between statin groups by Cox regression model adjusting for age, sex, year treatment started, dose, alcohol consumption, smoking, body mass index and comorbid conditions.

**Results:**

Moderate to severe hepatotoxicity [bilirubin >60μmol/L, AST or ALT >200U/L or alkaline phosphatase >1200U/L] developed in 71 patients on atorvastatin versus 101 on simvastatin. Adjusted hazard ratio [AHR] for all atorvastatin relative to simvastatin was 1.9 [95% confidence interval 1.4–2.6]. High dose was classified as 40–80mg daily and low dose 10–20mg daily. Hepatotoxicity occurred in 0.44% of 4075 patients on high dose atorvastatin [HDA], 0.07% of 72,336 on low dose atorvastatin [LDA], 0.09% of 44,675 on high dose simvastatin [HDS] and 0.05% of 119,732 on low dose simvastatin [LDS]. AHRs compared to LDS were 7.3 [4.2–12.7] for HDA, 1.4 [0.9–2.0] for LDA and 1.5 [1.0–2.2] for HDS.

**Conclusions:**

The risk of hepatotoxicity was increased in the first six months of atorvastatin compared to simvastatin treatment, with the greatest difference between high dose atorvastatin and low dose simvastatin. The numbers of events in the analyses were small.

## Introduction

In the UK the National Institute for Health and Care Excellence [NICE] has recommended that primary prophylaxis with atorvastatin should now be considered for all individuals between the age of 30 to 84 years with a 10 year cardiovascular risk score of 10% or greater [1]. This has been estimated to include about 25% of this population and would constitute mass medication of healthy individuals [2]. This guidance has stimulated controversy over possible adverse effects of statins in a healthy population [3] and the observation that more information is required. Statins are widely used and generally well tolerated. Adverse effects include myopathy, an increased incidence of diabetes and an increase in serum transaminases [4].

Raised serum transaminases of hepatic origin are seen in about 1–3% of individuals treated with statins but occasional more severe reactions can occur, with possible autoimmune hepatitis, resulting in hospitalisation and rare deaths [5–21]. Most studies looking at drug-induced liver disease related to statins have relied on results from registration clinical studies or self-reported adverse drug event registers [22–27] which may lead to under-reporting of events. The UK General Practice Research Database [GPRD] offered an opportunity to retrospectively examine all patients within a large sample population commenced on statin therapy in a “real world setting” and allowed examination of the true incidence of severe biochemical abnormalities associated with the use of atorvastatin. The GPRD became the Clinical Practice Research Datalink [CPRD] in March 2012. Simvastatin was selected as the comparator to atorvastatin as it is the most commonly prescribed statin in the UK and has few reports of hepatotoxicity. Also the patient population prescribed the two drugs should be comparable eliminating many other possible variables.

## Patients and Methods

### Study Design

A retrospective cohort study of the UK GPRD was undertaken comparing two groups of patients commenced on atorvastatin or simvastatin between the 10-year period 1997 to 2006 with regard to the incidence of hepatic reactions within this group.

### Study population

A total of 245,025 patients were identified on the GPRD who had a first prescription [index date] for atorvastatin or simvastatin during the period 1^st^ of January 1997 to the 31^st^ of December 2006. Patients had to be permanently registered with the practice for at least twelve months prior to the index date with no record of liver disease, alcohol-related diagnosis, statin prescription or biochemical evidence of liver dysfunction prior to index date Table 1. Further exclusion was made of patients under 18 years of age [n = 79] and patients with self-reported alcohol consumption > 50 units/week [n = 1571]. To avoid counting prevalent hepatotoxicity, all patients with a record of any of a list of selected liver-related conditions were censored 90 days preceding the date of that condition code. As a result 118 patients were not included in the final analysis. A further 1234 patients were also not included for having a code recording an alcohol-related diagnosis. This resulted in a final analysis population of 242,023 patients with 165,188 patients prescribed simvastatin and 76,835 patients prescribed atorvastatin. High dose was classified as 40–80mg daily and low dose 10–20mg daily.

**Table 1:** Exclusion criteria for patient selection prior to index date of statin prescription.

Alcohol related diagnosis
Pre-existing liver disease
○ acute or chronic viral hepatitis
○ autoimmune liver disease, primary biliary cirrhosis, autoimmune hepatitis, primary sclerosing cholangitis
○ extra-hepatic biliary obstruction
○ metastatic liver disease
○ haemochromatosis
○ liver biopsy or transplant
Abnormal liver function:
○ bilirubin levels >25 μmol/L
○ AST levels >120 U/L
○ ALT levels >120 U/L
○ Alkaline Phosphatase levels >300 U/L
Previous prescription of any statin

These patients were followed up until they either reached the end of the study period, changed or stopped their prescribed statin, altered the dose of statin moving between low and high dose groups, transferred out of their GP practice, died, or were within 90 days of having a code recording liver disease that could not be drug induced [a post-exclusion event]. A patient’s ‘on drug’ exposure period [defined by recorded prescriptions] ended either on the day preceding the first change of statin or dose, or, if the statin was stopped altogether, 28 days after the date of the last statin prescription [because 28 days was the most common gap between prescriptions]. This study reports data for the on-drug period only. Only 11% of patients on simvastatin and 26% of patients on atorvastatin were censored due to a change to the higher dose group during the six month period we analysed after initiation of therapy. Several potential confounding factors such as age, sex, serum cholesterol, BMI, calendar year of first prescription and comorbidity and baseline patient characteristics are shown in Table 2.

**Table 2:** Baseline characteristics of patients on simvastatin and atorvastatin grouped by statin dose.

		Data Complete	Simvastatin	Simvastatin	Atorvastatin	Atorvastatin
Dose			10–20 mg	40–80 mg	10–20 mg	40–80 mg
**Total number**	**242023**	100%	120367	44821	72741	4094
**Age [years]**	**Mean [SD]**	100%	66 [12]	65 [12]	64 [12]	62 [13]
**Sex**	**Male**	100%	61299 [50.9%]	24659 [55%]	37341 [51.3%]	2355 [57.55%]
**BMI**	**Mean [SD]**	95%	27.9 [5.2]	28.3 [5.5]	28.3 [5.3]	28.2 [5.4]
**Serum cholesterol [mmol/L]**	**Mean [SD]**	85%	6.3 [1.1]	6.2 [1.2]	6.5 [1.2]	6.5 [1.6]
**Alcohol [U/wk]**	**Mean [SD]**	55%	8.8 [9.5]	9.3 [9.9]	8.9 [9.6]	10 [10.4]
**Index year**	**Mean [SD]**	100%	2003.5 [2.6]	2005.1 [1.4]	2003.2 [2.1]	2004.9 [1.5]
**Length of follow-up [years]**	**Mean [SD]**	100%	1.9 [2.0]	1.3 [1.2]	2.4 [1.9]	1.4 [1.3]
**Medical history recorded**	**Myocardial Infarct**	*	9969 [8.3%]	5146 [11.5%]	5516 [7.6%]	1088 [26.6%]
	**Coronary heart disease**	*	30158 [25.1%]	8962 [20%]	16953 [23.3%]	1076 [26.3%]
	**Cerebrovascular disease**	*	12183 [10.1%]	5459 [12.2%]	6973 [9.6%]	325 [7.9%]
	**Peripheral vascular disease**	*	3102 [2.6%]	1097 [2.4%]	1941 [2.7%]	100 [2.4%]
	**Diabetes mellitus**	*	25755 [21.4%]	9807 [21.9%]	18807 [25.8%]	636 [15.5%]
	**Hypertension**	*	62470 [51.9%]	22559 [50.3%]	36190 [49.7%]	1696 [41.4%]
	**MI within 30 days prior to index date**	*	3587 [3%]	3121 [7%]	1990 [2.7%]	808 [19.7%]
	**Any of MI, CHD, CVD, PVD**	*	44116 [36.6%]	16872 [37.6%]	25150 [34.6%]	2076 [50.7%]

SD—standard deviation.

*Medical history data completeness could not be calculated because non-occurrence could not be distinguished from non-recording of a condition

The General Practice Research Database group has obtained blanket ethics approval for observational studies using the General Practice Research Database and studies using the General Practice Research Database that result in publication require prior approval by the Independent Scientific Advisory Committee, Medicines & Healthcare products Regulatory Agency, 151 Buckingham Palace Road, London, SW1W 9SZ, UK England.

The study was approved by General Practice Research Database research ethics committee: Independent Scientific Advisory Committee Reference Number Protocol No. 08_039 [Applicant Dr Peter Mills].

The data from General Practice Research Database is rigorously checked prior to release to ensure it is anonymized with no patient identifiers. We did not approach patients directly for consent as the data was supplied with licensed consent to use the data given by the Secretary of State for Health.

### Definition of outcomes

Hepatotoxicity was defined as first evidence of abnormal liver biochemistry with either a serum bilirubin ≥ 40 μmol/l, serum aspartate aminotransferase [AST] or alanine aminotransferase [ALT] ≥ 120 U/l [3 x ULN] or serum alkaline phosphatase [ALP] > 2 x ULN or ≥ 600 U/l. Our cut-off values were primarily based upon local laboratory levels and we used 3 times the upper limit of normal for AST/ALT, 2 x the upper limit of normal for ALP and a bilirubin of over 40 [higher than our laboratory upper limit of normal] to try to counter possible small differences in regional definitions of normal/abnormal enzyme levels.

Our primary endpoint was an episode of at least moderate hepatotoxicity which was a serum bilirubin ≥ 60 μmol/l, serum AST or ALT ≥ 200 U/l [5 x ULN] or serum alkaline phosphatase > 4 x ULN or ≥ 1200 U/l within the first six months of follow-up after initiation of therapy. Other secondary endpoints defined as mild or severe liver dysfunction are categorised in Table 3.

**Table 3:** Categorisation of abnormal liver biochemistry recorded after index date.

Hepatotoxicity	Mild	Moderate	Severe
Bilirubin [μmol/L]	40–59	60–79	≥80
AST or ALT [U/L]	120–199	200–399	≥400
Alkaline Phosphatase	2–4 x ULN	4–6 x ULN	>6 x ULN
[U/L]	600–1199	1200–1799	≥1800

In patients with laboratory evidence of abnormal hepatic function as above, the results were divided into three subcategories. Any one laboratory result at any point will qualify for inclusion in that category.

### Data Analysis

The risk of reaching the primary endpoint was compared between patients being prescribed atorvastatin and simvastatin by estimating hazard ratios with 95% confidence intervals [CI] from Cox proportional hazards regression models. We investigated the possibility that a difference in risk between atorvastatin and simvastatin might depend upon statin dose by testing for an interaction between prescribed statin and dose [high [40 to 80 mg] or low [10 to 20 mg]] and estimating hazard ratios for high- and low-dose atorvastatin and high dose simvastatin relative to low dose simvastatin.

Atorvastatin has been reported to be more effective than simvastatin at lowering cholesterol [28], and higher doses are more effective than lower doses [29], so patients with more severe vascular disease, higher cholesterol levels or drug-resistant hypercholesterolaemia are more likely to be prescribed atorvastatin and/or a high dose. We therefore expected to observe an association between both prescribed statin, dose and risk of hepatotoxicity even in the absence of a causal link [confounding by indication]. The statin-hepatotoxicity association is also expected to be confounded by secular trends in statin prescribing patterns. We mitigated the impact of confounders by adjusting hazard ratios for age in years [categorical: 18–50, 51–60, 61–70, 71–80, 81–90, ≥ 91], sex, year of first prescription [categorical: 1997–2001, 2002–2003, 2004–2006; boundaries were chosen to give approximately equal numbers of patients in each category], self-reported alcohol consumption in units/week [categorical: 0, 1–7, 8–14, 15–21, ≥ 22], smoking habit [never, former, current], serum cholesterol level in mmol/L [categorical: < 5, 5–5.99, 6–6.99, ≥ 7], BMI in kg/m^2^ [categorical: < 25, 25–29.9, ≥ 30], histories of MI, coronary heart disease, cerebrovascular disease, diabetes and hypertension and time of MI prior to entry to the study [categorical: 0–30 days, 31 days-1 year, 1+ years, no history].

In addition to quantifying relative risks as hazard ratios, we estimated excess risk in terms of absolute numbers of events by two methods. First, we estimated the number of events per 1000 patient-years of follow-up in each group as the predicted probability of a patient suffering an event within six months [1 –*S*, where *S* is the probability of not experiencing an event within six months] multiplied by 2000. Second, we estimated the number of patients that would need to be treated [NNT] to predict an additional instance of hepatotoxicity. The NNT statistic was calculated from the survival probabilities following equation 1 of Altman and Andersen [30]. It is equivalent to the number needed to treat to cause harm to one patient compared with control in a clinical trial, but differs in interpretation. For both analyses, survival probabilities were estimated from a Cox regression model adjusted to a “typical” patient, that is, one who falls into the most frequent category of each adjustment covariate [male, aged 61–70 years, never a smoker, alcohol consumption not known, serum cholesterol 6–6.99 mmol/L, BMI 25–29.9 kg/m^2^, no history of MI, CHD or CVD, a history of hypertension and index year 2004–2006]. 95% confidence intervals for both statistics were estimated from 20,000 bootstrap replicates.

Because excluding patients with missing alcohol, serum cholesterol or BMI would have reduced the size of the available data by 53%, we chose instead to fit missing alcohol, serum cholesterol and BMI data as additional categories. We assessed the sensitivity of the results to this approach by re-estimating hazard ratios from the reduced data set. Smoking data could not be dealt with in this way because too few data were missing [N = 1205] to allow estimation of a hazard ratio, so patients with missing smoking data were excluded.

In an observational study it is never possible to adjust for all of the differences between patients that affect outcome. We therefore expected the adjusted hazard ratios to be biased by residual confounding. For example, patients are at increased risk of hepatotoxicity immediately following a myocardial infarction, and such patients are more likely to be prescribed atorvastatin and a high dose Table 2. We investigated this potential source of residual confounding by re-estimating the adjusted hazard ratios after the exclusion of patients with a myocardial infarction in the 30 days prior to first statin prescription. Another potential source of residual confounding is the fact that where no clinical history is recorded we do not know whether this is because the patient has no history or because the history has not been recorded in GPRD. We assessed the sensitivity of the adjusted hazard ratios to missing clinical history by excluding patients with no history of any of myocardial infarction, coronary heart disease, cerebrovascular disease or peripheral vascular disease. Where the numbers in the excluded subgroup were sufficient, we tested for a significant difference in the hazard ratios between the included and excluded subgroups.

Stratification of outcomes was performed according to statin dose to examine whether there was a dose-related effect in determining adverse outcome. All statistical analyses were performed using R version 2.13.0 [31].

## Results

### Moderate to severe hepatotoxicity for simvastatin and atorvastatin

71 [0.09%] of all 76411 patients on atorvastatin developed moderate or severe hepatotoxicity versus 101 [0.06%] of all 164407 patients on simvastatin in the first six months on treatment Table 4. The adjusted hazard ratio for atorvastatin relative to simvastatin was 1.9 [95% confidence interval 1.4–2.6; p<0.001].

**Table 4:** Hazard ratio [HR] estimates [95% CI] for significant lab events associated with atorvastatin during the first 6 months therapy on treatment, relative to simvastatin.

Outcome	N		Prescribed statin		P
			Simvastatin	Atorvastatin	
			164407	76411	
Hepatotoxicity	240818	N [%]	407 [0.25%]	245 [0.32%]	
All grades		Unadj. HR	1 [ref]	1.2 [1.0, 1.4]	0.02
		Adj. HR	1 [ref]	1.4 [1.2, 1.7]	<0.001
Hepatotoxicity	240818	N [%]	101 [0.06%]	71 [0.09%]	
Moderate to severe		Unadj. HR	1 [ref]	1.4 [1.0, 1.9]	0.03
		Adj. HR	1 [ref]	1.9 [1.4, 2.6]	<0.001

1205 [0.5%] patients with missing smoking data were excluded.

N = number

### Moderate to severe hepatotoxicity in high versus low dose statin groups

Hepatotoxicity was more common in patients on 40–80 mg than 10–20 mg of either statin [adjusted hazard ratio: 2.1; 95% CI 1.5–3.0; p<0.001] Table 5. The strength of the association with prescribed statin was stronger at high doses [interaction p<0.001], with hepatotoxicity being most common in patients on high dose atorvastatin. Moderate to severe hepatotoxicity occurred in 18 [0.44%] of 4075 high dose atorvastatin patients in comparison to 53 [0.07%] of 72336 patient on low dose atorvastatin, in 39 [0.09%] of 44675 patients on high dose simvastatin and 62 [0.05%] of 119732 patients on low dose simvastatin. Adjusted hazard ratios calculated relative to low dose simvastatin were 7.3 [4.2–12.7; p<0.001] for high dose atorvastatin, 1.4 [0.9–2.0; p = 0.095] for low dose atorvastatin and 1.5 [1.0–2.2; p = 0.068] for high dose simvastatin.

**Table 5:** Hazard ratio [HR] estimates [95% CI] for significant lab events associated with prescribed statin and dose during the first 6 months therapy on treatment, relative to low dose simvastatin.

Outcome	N		Prescribed statin and dose				
			Simvastatin	Simvastatin	Atorvastatin	Atorvastatin	P
			10–20 mg	40–80 mg	10–20 mg	40–80 mg	
			119732	44675	72336	4075	
Hepatotoxicity	240818	N [%]	258 [0.22%]	149 [0.33%]	205 [0.28%]	40 [0.98%]	
		Unadj. HR	1 [ref]	1.6 [1.3, 2.0], P<0.001	1.2 [1.0, 1.5], P = 0.02	4.6 [3.3, 6.4], P<0.001	<0.001
All grades		Adj. HR	1 [ref]	1.3 [1.0, 1.5], P = 0.03	1.3 [1.0, 1.5], P = 0.015	3.3 [2.3, 4.7], P<0.001	<0.001
Hepatotoxicity	240818	N [%]	62 [0.05%]	39 [0.09%]	53 [0.07%]	18 [0.44%]	
Moderate to severe		Unadj. HR	1 [ref]	1.7 [1.2, 2.6], P = 0.007	1.3 [0.9, 1.9], P = 0.13	8.6 [5.1, 14.6], P<0.001	<0.001
		Adj. HR	1 [ref]	1.5 [1.0, 2.2], P = 0.07	1.4 [0.9, 2.0], P = 0.095	7.3 [4.2, 12.7], P<0.001	<0.001

1205 [0.5%] patients with missing smoking data were excluded.

N = number

The distribution of liver function test severity levels among the 172 patients who experienced the primary endpoint of at least moderate hepatotoxicity with at least one of the four tests within 6 months of initiation of statin therapy is given in S1 Table.

### Numbers needed to harm

**Fig 1:**
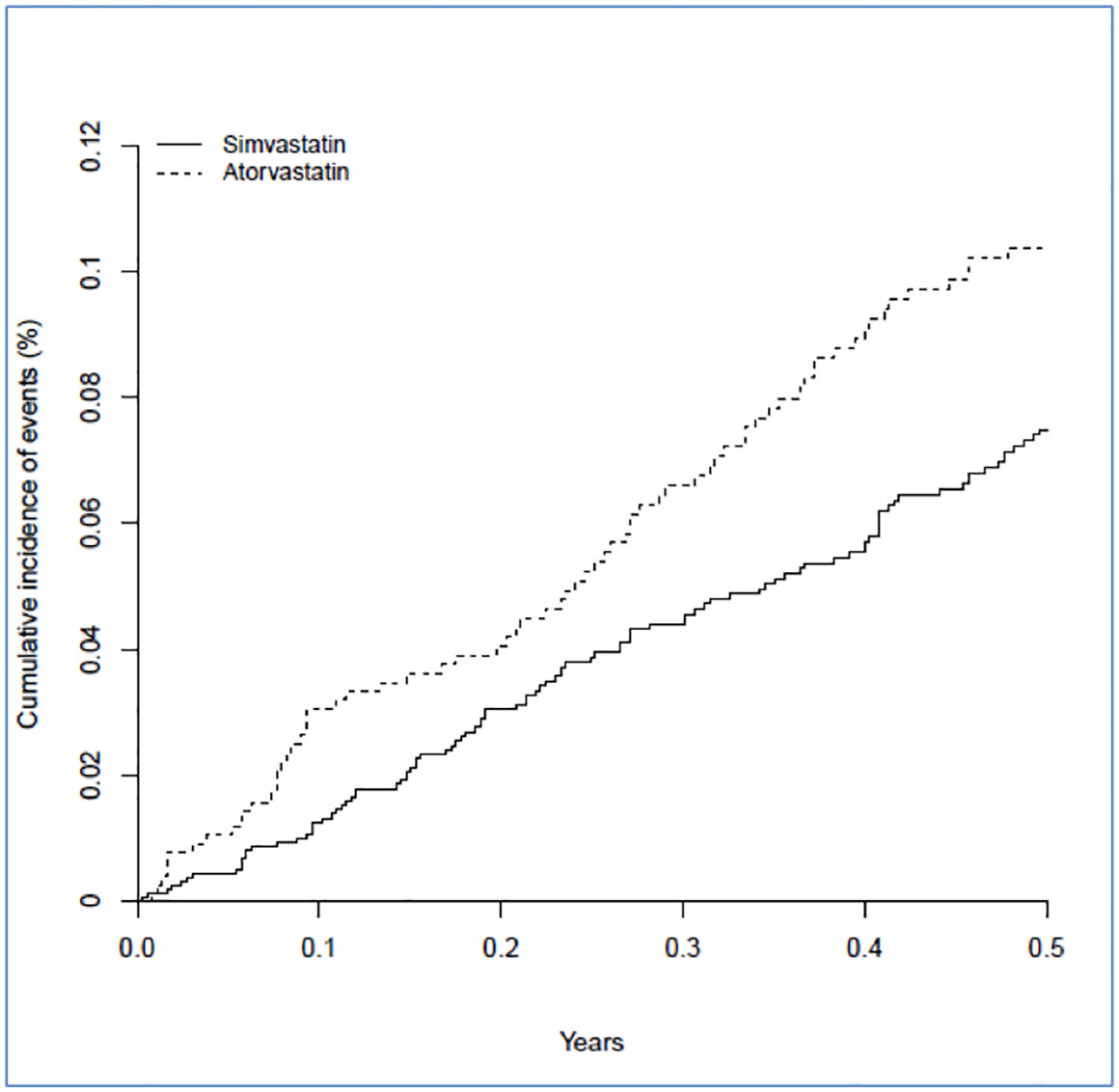
Cumulative incidence of moderate-severe hepatotoxicity for atorvastatin and simvastatin [all doses] over 6 months after index dose.

**Fig 2:**
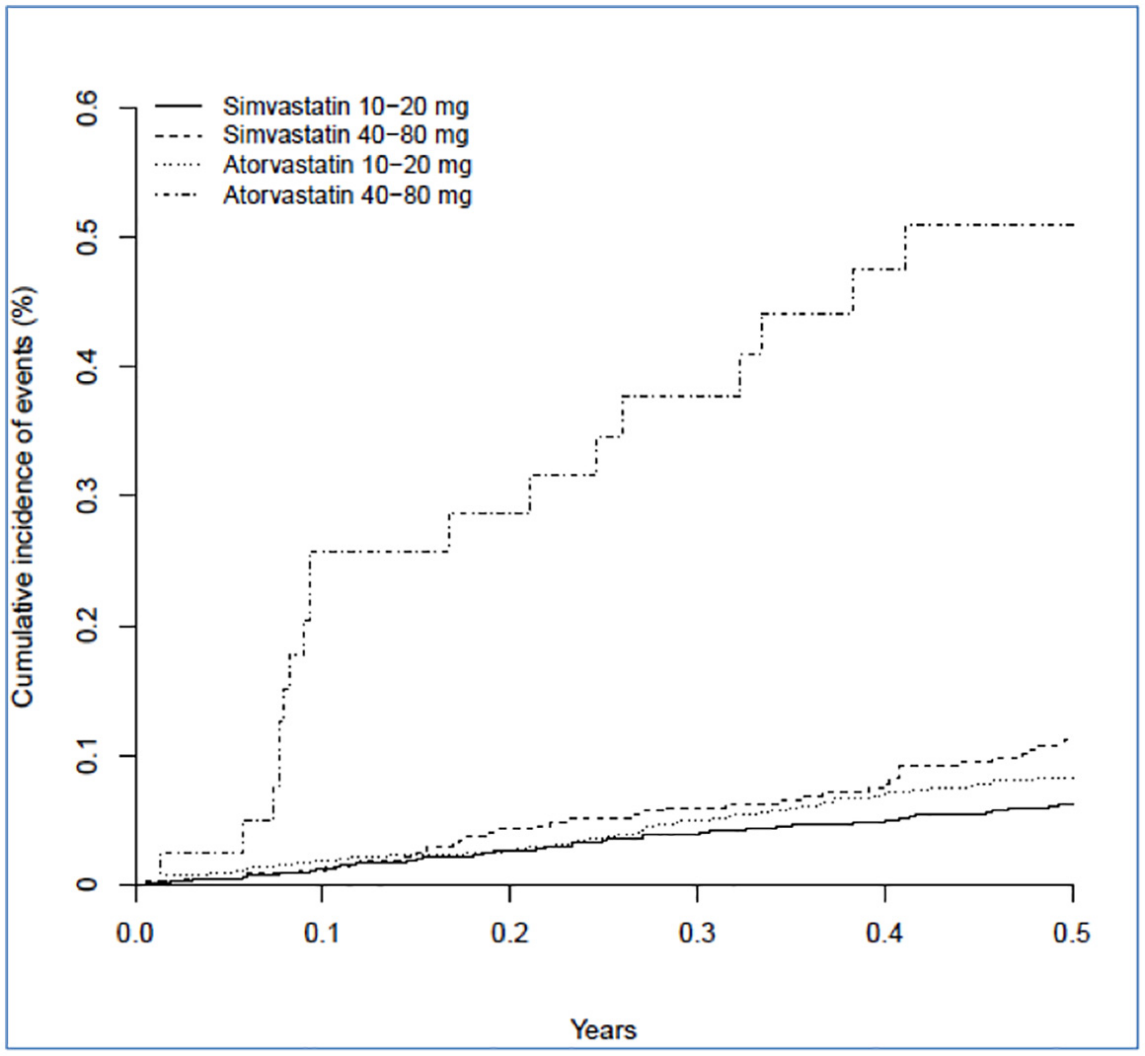
Cumulative incidence of moderate-severe hepatotoxicity for atorvastatin and simvastatin [high and low dose groups] over 6 months after index dose.

The number of instances of moderate to severe hepatotoxicity per 1000 patient-years of follow-up was 0.9 [0.4–1.6] for low-dose simvastatin, 1.3 [0.6–2.3] for high-dose simvastatin, 1.2 [0.6–2.1] for low-dose atorvastatin and 6.5 [2.8–11.9] for high-dose atorvastatin. The excess risk of moderate to severe hepatotoxicity in the high dose atorvastatin group relative to low dose simvastatin translates to one additional event in six months per 359 [95% CI 188–912] patients treated. Cumulative incidence for moderate to severe hepatotoxicity for both atorvastatin and simvastatin and also for high versus low dose groups over the first six months after index dose is illustrated in Figs 1 and 2.

### Effect/absence of significant vascular events on hepatotoxicity

The hazard ratios were not substantially affected by excluding patients suffering myocardial infarction in the 30 days prior to statin treatment [S2 Table], although we could not formally test this due to the low numbers of patients reaching an endpoint in the excluded subgroup [14/9438 patients with recent MI suffered hepatotoxicity]. Similarly, excluding patients with no history of vascular disease had no significant effect on the hazard ratios [interaction p = 0.355; S3 Table].

## Discussion

Hepatotoxicity was defined by laboratory test to include either a serum bilirubin >60 μmol/L, AST or ALT >200 U/L or alkaline phosphatase >1200 U/L. This increased risk was predominantly experienced by patients on high dose atorvastatin, although the numbers of events in the analyses were small. 71 events [0.09%] were recorded on atorvastatin in comparison to 101 events [0.06%] on simvastatin with an adjusted hazard ratio of 1.9 for atorvastatin in comparison to simvastatin.

There have been 40 cases in the published literature of significant hepatotoxicity due to statins [12] with one of the larger case series, suggesting concern over atorvastatin, published by our group [5]. However, there is ongoing debate in the literature as to whether such concern over hepatotoxicity due to statins has any foundation with the major cardiovascular trials often quoted as demonstrating no safety concerns. It has been noted that the large trials devised for evaluating morbidity and mortality benefits of statins in treating cardiovascular disease were not designed with the intent of assessing the risk of drug-induced liver injury [DILI]. Similar rates of abnormal liver function tests were observed between placebo and treatment arms of large statin trials including EXCEL, the 4S study, the MIRACL study and WOSCOPS [32–35].

Since the large clinical statin trials, there have been studies looking specifically at the safety of statins with respect to drug-induced liver injury. Hepatic injury due to statins was reported by a group who analysed episodic reports of adverse drug reactions sent to the Swedish Adverse Drugs Reactions Advisory Committee between 1988 and 2010 [24]. Their definition of hepatotoxicity included transaminases five times the upper limit of normal or alkaline phosphatase at twice the upper limit of normal. They found 73 patients with hepatotoxicity of whom 30 [41%] were taking atorvastatin and 28 [38%] simvastatin with two deaths and one requirement for liver transplant. They demonstrated overall statin-related hepatotoxicity in 1.6/100,000 patient-years, with 2.9 events/100,000 patient-years for atorvastatin and 0.9 events/100,000 patient-years for simvastatin. Our systematic study demonstrated a more frequent rate of moderate to severe hepatotoxicity with 0.9 events/1000 patient-years for low-dose simvastatin [i.e. 90 events per 100000 patient-years compared to the rate of 0.9 events per 100000 patient years seen for simvastatin in the Swedish study], 1.3 events/1000 patient-years for high-dose simvastatin, 1.2 events/1000 patient-years for low-dose atorvastatin and 6.5 events/1000 patient-years for high-dose atorvastatin. The excess risk of moderate to severe hepatotoxicity for a patient on high dose atorvastatin group compared to a patient on low dose simvastatin translated to one additional hepatotoxic event in six months for every 359 patients treated. Our study had a significantly higher rate of events than seen in the Swedish study and may reflect our different methods and definitions. The Swedish study, as do most adverse event registries, relied on the healthcare reporting of adverse events [and could feasibly under-report the frequency of statin-related events] and cannot be compared directly with elevated liver function tests in routine testing recorded on the GPRD. They also calculated the incidence of statin-induced hepatotoxicity based on the total statin prescription data in Sweden during the study period whereas we report the prescription data only for the subjects in a well defined GPRD cohort. One final point of note is that the Swedish paper did include causality assessment by means of the RUCAM score which led to exclusion of some patients due to incomplete clinical data whereas we did not perform such an assessment.

Previous studies of drug-related adverse events have suggested that statins contribute to between approximately 1 to 3% of all drug-induced liver injury. A study by Bjornsson et al [25] reported on 784 cases of drug-induced liver injury and suspected statins in 8 [4 atorvastatin, 4 simvastatin] of these cases with two of the eight patients going to liver transplant and death respectively. Andrade et al [26] prospectively examined 461 cases of drug-induced liver injury reported to the Spanish Hepatotoxicity Registry, implicating statins in 18 cases [3%] and a similar proportion of drug-induced liver injury [3.4%] was reported by Chalasani et al [27].

Previous studies have shown severe adverse hepatic events related to statins to be infrequent. The Acute Liver Failure Study Group reported on 133 prospectively collected cases of acute liver failure due to drug-induced liver injury between 1998 and 2010 [36]. 2 patients [1.5%] had taken atorvastatin, 2 had taken simvastatin and 2 had taken the now discontinued cerivastatin. Interestingly, high ANA titres were seen in cases due to cerivastatin and simvastatin, suggesting a possible drug-induced autoimmune phenomenon. Other groups have also highlighted an apparent link between statins and autoimmune hepatitis [37–42].

A comparative safety analysis of 49 trials involving 14236 patients looked at the effect of dosage on adverse events due to atorvastatin [43]. The incidence of any ALT or AST elevations greater than 3 times the upper limit of normal was 0.6% in the atorvastatin 10 mg group, 0.6% in the placebo group and 3.3% in the atorvastatin 80 mg group. The positive association observed in this study between atorvastatin dose and hepatotoxicity incidence mirrors our own findings.

A recent update by the Statin Liver Safety Task Force concluded that recorded hepatotoxicity due to all statins was still a rare event The group reported that the FDA had conducted several post-marketing reviews of statins and hepatotoxicity from 2000 to 2009 using the Agency’s Adverse Event Reporting System [AERS] database. The reporting of statin-associated serious liver injury to the AERS database was extremely low [reporting rate of less than two per one million patient-years] [44]. This discrepancy with our own study may reflect the potential under-reporting of adverse events in registries discussed previously in our paper.

One of the weaknesses of our study was the high frequency of missing data due to variation among clinicians in recording data (Table 2). However, we assessed the impact of missing data using sensitivity analyses. Another weakness of retrospective GPRD studies is that there is inevitably variation in reference ranges for laboratory values between different practices. To address this we devised a set of laboratory criteria to reflect severe levels of liver injury. The degree of liver dysfunction to qualify as moderate hepatotoxicity was quite stringent to ensure that the large numbers with mild elevation of serum transaminases commonly seen with statins were not observed as an end-point. Another final point of note is that our events per patient-years calculation was based upon events recorded within six months of drug initiation. We analysed the initial six months as previous case series have demonstrated that the majority of idiosyncratic liver reactions are seen in the initial period after commencement of statin [12]. Given that our mean length of follow-up was up to 2.4 years for low dose atorvastatin, 1.9 years for low dose simvastatin, 1.4 years for high dose atorvastatin and 1.3 years for high dose simvastatin, any events per patient-year calculation based upon all observed follow-up may have been influenced by this variation in therapy duration between the different statin-dose groups.

In conclusion, patients on atorvastatin were found to have an overall increased risk of hepatotoxicity in comparison to those on simvastatin. This increased risk was predominantly experienced by patients on high dose atorvastatin with an incidence of biochemical hepatotoxicity of 6.5 per 1,000 patient-years. This risk of measured hepatic events was significantly higher than previously reported studies which were based upon healthcare reporting and may reflect under-reporting in such systems. Low dose statin therapy appears to be relatively safer than high dose atorvastatin in our study. However the absolute difference in risk is small.

## Supporting Information

S1 Table Distribution of liver function test severity levels among the 172 patients who experienced the primary endpoint of at least moderate hepatotoxicity with at least one of the four tests within 6 months of initiation of statin therapy. (DOCX)

S2 Table Hazard ratio [HR] estimates [95% CI] for significant laboratory events associated with statin-dose combinations during the first 6 months therapy, relative to low dose simvastatin. 9438 patients with MI within 30 days prior to therapy initiation were excluded.
(DOCX)

S3 Table Hazard ratio [HR] estimates [95% CI] for significant lab events associated with statin-dose combinations during the first 6 months therapy, relative to low dose simvastatin. Only patients with a history of MI, CHD, CVD or PVD were included.
(DOCX)

## Abbreviations

AHR: adjusted hazard ratio
HDA: high dose atorvastatin
LDA: low dose atorvastatin
HDS: high dose atorvastatin
LDS: low dose simvastatin
NICE: National Institute for Health and Care Excellence
GPRD: General Practice Research Database
CI: confidence intervals
NNT: numbers needed to treat
DILI: drug induced liver injury
AST: aspartate aminotransferase
ALT: alanine aminotransferase
ALP: alkaline phosphatase
