# Supplementary material for: High Dose Atorvastatin Associated with Increased Risk of Significant Hepatotoxicity in Comparison to Simvastatin in UK GPRD Cohort
Source: PLoS One. 2016 Mar 16;11(3):e0151587. doi: 10.1371/journal.pone.0151587 (PMC4794178; doi:10.1371/journal.pone.0151587)
Supplement: S1 Table — (DOCX) [file pone.0151587.s001.docx]

|  |  | Total | Prescribed statin and dose | | | |
| --- | --- | --- | --- | --- | --- | --- |
|  |  |  | Simvastatin 10-20 mg | Simvastatin 40-80 mg | Atorvastatin 10-20 mg | Atorvastatin 40-80 mg |
| Bilirubin | N_OBS_ (N_MISSING_) Normal Mild Moderate Severe | 157 (15) 108 (69%) 23 (15%) 8 (5%) 18 (11%) | 58 (4) 38 (66%) 10 (17%) 2 (3%) 8 (14%) | 32 (7) 24 (75%) 5 (16%) 2 (6%) 1 (3%) | 50 (3) 32 (64%) 7 (14%) 2 (4%) 9 (18%) | 17 (1) 14 (82%) 1 (6%) 2 (12%) 0 (0%) |
| AST | N_OBS_ (N_MISSING_) Normal Mild Moderate Severe | 133 (39) 22 (17%) 9 (7%) 72 (54%) 30 (23%) | 42 (20) 12 (29%) 1 (2%) 19 (45%) 10 (24%) | 34 (5) 3 (9%) 3 (9%) 21 (62%) 7 (21%) | 43 (10) 6 (14%) 4 (9%) 23 (53%) 10 (23%) | 14 (4) 1 (7%) 1 (7%) 9 (64%) 3 (21%) |
| ALT | N_OBS_ (N_MISSING_) Normal Mild Moderate Severe | 52 (120) 11 (21%) 9 (17%) 21 (40%) 11 (21%) | 23 (39) 7 (30%) 2 (9%) 8 (35%) 6 (26%) | 11 (28) 2 (18%) 2 (18%) 6 (55%) 1 (9%) | 13 (40) 2 (15%) 3 (23%) 5 (38%) 3 (23%) | 5 (13) 0 (0%) 2 (40%) 2 (40%) 1 (20%) |
| ALP | N_OBS_ (N_MISSING_) Normal Mild Moderate Severe | 162 (10) 75 (46%) 39 (24%) 27 (17%) 21 (13%) | 61 (1) 24 (39%) 20 (33%) 10 (16%) 7 (11%) | 31 (8) 25 (81%) 1 (3%) 2 (6%) 3 (10%) | 53 (0) 23 (43%) 12 (23%) 11 (21%) 7 (13%) | 17 (1) 3 (18%) 6 (35%) 4 (24%) 4 (24%) |

**S1 Table:** Distribution of liver function test severity levels among the 172 patients who experienced the primary endpoint of at least moderate hepatotoxicity with at least one of the four tests within 6 months of initiation of statin therapy.

For each test, data are presented as the number of test results available (N_OBS)_ and missing (N_MISSING_) and the number (%) of patients at each severity level, overall and within groups defined by their prescribed statin and dose.

Normal, mild, moderate and severe liver function test levels were defined using the thresholds in Table 3.

AST: aspartate aminotransferase, ALT: alanine aminotransferase, ALP: alkaline phosphatase
