# Supplementary material for: High Dose Atorvastatin Associated with Increased Risk of Significant Hepatotoxicity in Comparison to Simvastatin in UK GPRD Cohort
Source: PLoS One. 2016 Mar 16;11(3):e0151587. doi: 10.1371/journal.pone.0151587 (PMC4794178; doi:10.1371/journal.pone.0151587)
Supplement: S2 Table — 9438 patients with MI within 30 days prior to therapy initiation were excluded. (DOCX) [file pone.0151587.s002.docx]

|  | | | | | | | |
| --- | --- | --- | --- | --- | --- | --- | --- |
| **Outcome** | **N** |  | **Statin-dose combination** | | | | **P** |
|  |  |  | **Simvastatin 10-20 mg**  116185 | **Simvastatin 40-80 mg**  41568 | **Atorvastatin 10-20 mg**  70359 | **Atorvastatin 40-80 mg**  3268 |  |
| Hepatotoxicity All grades | 231380 | N (%) Unadj. HR Adj. HR | 247 (0.21%) 1 (ref) 1 (ref) | 134 (0.32%) 1.6 (1.3, 1.9), P<0.001 1.3 (1.0, 1.6), P=0.025 | 196 (0.28%) 1.2 (1.0, 1.5), P=0.03 1.3 (1.0, 1.5), P=0.02 | 30 (0.92%) 4.4 (3.0, 6.4), P<0.001 3.5 (2.4, 5.1), P<0.001 | <0.001 <0.001 |
| Hepatotoxicity Moderate to severe | 231380 | N (%) Unadj. HR Adj. HR | 59 (0.05%) 1 (ref) 1 (ref) | 34 (0.08%) 1.7 (1.1, 2.5), P=0.02 1.4 (0.9, 2.2), P=0.10 | 51 (0.07%) 1.3 (0.9, 1.9), P=0.13 1.4 (1.0, 2.0), P=0.08 | 14 (0.43%) 8.6 (4.8, 15.4), P<0.001 8.0 (4.4, 14.6), P<0.001 | <0.001 <0.001 |

**S2 Table:** Hazard ratio (HR) estimates (95% CI) for significant lab events associated with statin-dose combinations during the first 6 months therapy after recruitment, relative to low dose simvastatin. 9438 patients with MI within 30 days prior to recruitment were excluded.

N = number
