# Supplementary material for: High Dose Atorvastatin Associated with Increased Risk of Significant Hepatotoxicity in Comparison to Simvastatin in UK GPRD Cohort
Source: PLoS One. 2016 Mar 16;11(3):e0151587. doi: 10.1371/journal.pone.0151587 (PMC4794178; doi:10.1371/journal.pone.0151587)
Supplement: S3 Table — Only patients with a history of MI, CHD, CVD or PVD were included. (DOCX) [file pone.0151587.s003.docx]

**S3 Table:** Hazard ratio (HR) estimates (95% CI) for significant lab events associated with statin-dose combinations during the first 6 months therapy after recruitment, relative to low dose simvastatin. Only patients with a history of MI, CHD, CVD or PVD were included.

| **Outcome** | **N** |  | **Statin-dose combination** | | | | **P** |
| --- | --- | --- | --- | --- | --- | --- | --- |
|  |  |  | **Simvastatin 10-20 mg**  43798 | **Simvastatin 40-80 mg**  16804 | **Atorvastatin 10-20 mg**  24994 | **Atorvastatin 40-80 mg**  2069 |  |
| Hepatotoxicity All grades | 87665 | N (%) Unadj. HR Adj. HR | 103 (0.24%) 1 (ref) 1 (ref) | 68 (0.40%) 1.8 (1.3, 2.4), P<0.001 1.2 (0.8, 1.6), P=0.37 | 80 (0.32%) 1.3 (1.0, 1.8), P=0.07 1.3 (1.0, 1.7), P=0.09 | 25 (1.21%) 5.4 (3.5, 8.3), P<0.001 3.3 (2.0, 5.3), P<0.001 | <0.001 <0.001 |
| Hepatotoxicity Moderate to severe | 87665 | N (%) Unadj. HR Adj. HR | 29 (0.07%) 1 (ref) 1 (ref) | 18 (0.11%) 1.7 (0.9, 3.0), P=0.09 1.2 (0.7, 2.3), P=0.49 | 16 (0.06%) 0.9 (0.5, 1.7), P=0.81 0.9 (0.5, 1.7), P=0.74 | 12 (0.58%) 9.2 (4.7, 18.0), P<0.001 7.2 (3.3, 15.8), P<0.001 | <0.001 <0.001 |

N = number
